# Quantitative CT analysis using functional imaging is superior in describing disease progression in idiopathic pulmonary fibrosis compared to forced vital capacity

**DOI:** 10.1186/s12931-018-0918-5

**Published:** 2018-11-06

**Authors:** J. Clukers, M. Lanclus, B. Mignot, C. Van Holsbeke, J. Roseman, S. Porter, E. Gorina, E. Kouchakji, K. E. Lipson, W. De Backer, J. De Backer

**Affiliations:** 1 Faculty of Medicine & Health Sciences, University of Antwerp (UAntwerpen), Universiteitsplein 1, 2610 Antwerpen, Belgium; 2 FluidDA nv, Groeningenlei 132, 2550 Kontich, Belgium; 3 FibroGen Inc., 409 Illinois Street, San Francisco, CA 94158 USA

**Keywords:** Functional respiratory imaging is superior in describing disease in IPF

## Abstract

**Background:**

Idiopathic pulmonary fibrosis (IPF) is chronic fibrosing pneumonia with an unpredictable natural disease history. Functional respiratory imaging (FRI) has potential to better characterize this disease. The aim of this study was to identify FRI parameters, which predict FVC decline in patients with IPF.

**Methods:**

An IPF-cohort (treated with pamrevlumab for 48 weeks) was retrospectively studied using FRI. Serial CT’s were compared from 66 subjects. Post-hoc analysis was performed using FRI, FVC and mixed effects models.

**Results:**

Lung volumes, determined by FRI, correlated with FVC (lower lung volumes with lower FVC) (*R*^*2*^ = 0.61, *p* < 0.001). A negative correlation was observed between specific image based airway radius (siRADaw) at total lung capacity (TLC) and FVC (*R*^*2*^ = 0.18, *p* < 0.001). Changes in FVC correlated significantly with changes in lung volumes (*R*^*2*^ = 0.18, *p* < 0.001) and siRADaw (*R*^*2*^ = 0.15, *p* = 0.002) at week 24 and 48, with siRADaw being more sensitive to change than FVC. Loss in lobe volumes (*R*^*2*^ = 0.33, *p* < 0.001), increasing fibrotic tissue (*R*^*2*^ = 0.33, *p* < 0.001) and airway radius (*R*^*2*^ = 0.28, *p* < 0.001) at TLC correlated with changes in FVC but these changes already occur in the lower lobes when FVC is still considered normal.

**Conclusion:**

This study indicates that FRI is a superior tool than FVC in capturing of early and clinically relevant, disease progression in a regional manner.

**Electronic supplementary material:**

The online version of this article (10.1186/s12931-018-0918-5) contains supplementary material, which is available to authorized users.

## Background

Idiopathic pulmonary fibrosis (IPF) is a fatal, chronic fibrosing interstitial pneumonia with a variable and unpredictable natural history [1–3]. Diagnosing IPF at an early stage enables more effective treatment and improvement of the long-term clinical outcome of this progressive debilitating disease [4–6]. Predicting prognosis is an important part of IPF management, but it remains difficult in individual patients with the current standard investigations as forced vital capacity (FVC) and high resolution computed tomography (HRCT) [7, 8].

FVC best predicts disease progression and mortality [9, 10]. It therefore serves as a primary endpoint in IPF, although it’s not a proven surrogate for mortality [11, 12]. A 2–6% change in predicted FVC has been proposed as the minimal clinical important difference [9], and where 10% decline in absolute FVC correlated well with mortality [10]. Due to weak signal-to-noise ratios [13], FVC is not able to pick up small changes in progressing fibrosis in these patients. In future studies, where patients are treated with (combination of) antifibrotic drugs, its use as a clinical endpoint is less compelling since FVC decline is impacted by this therapy [14–16].

The disease stage, as measured by HRCT, has been correlated to lung function measurements [17, 18]. In recent years quantitative computer-derived CT (qCT) variables have been studied in IPF, and have been shown to be superior predictors of mortality compared to any visually scored CT parameter (e.g. extent of fibrosis) [19, 20]. Despite these advances in the field, no radiological marker is widely accepted as a biomarker in IPF [21].

**Fig. 1:**
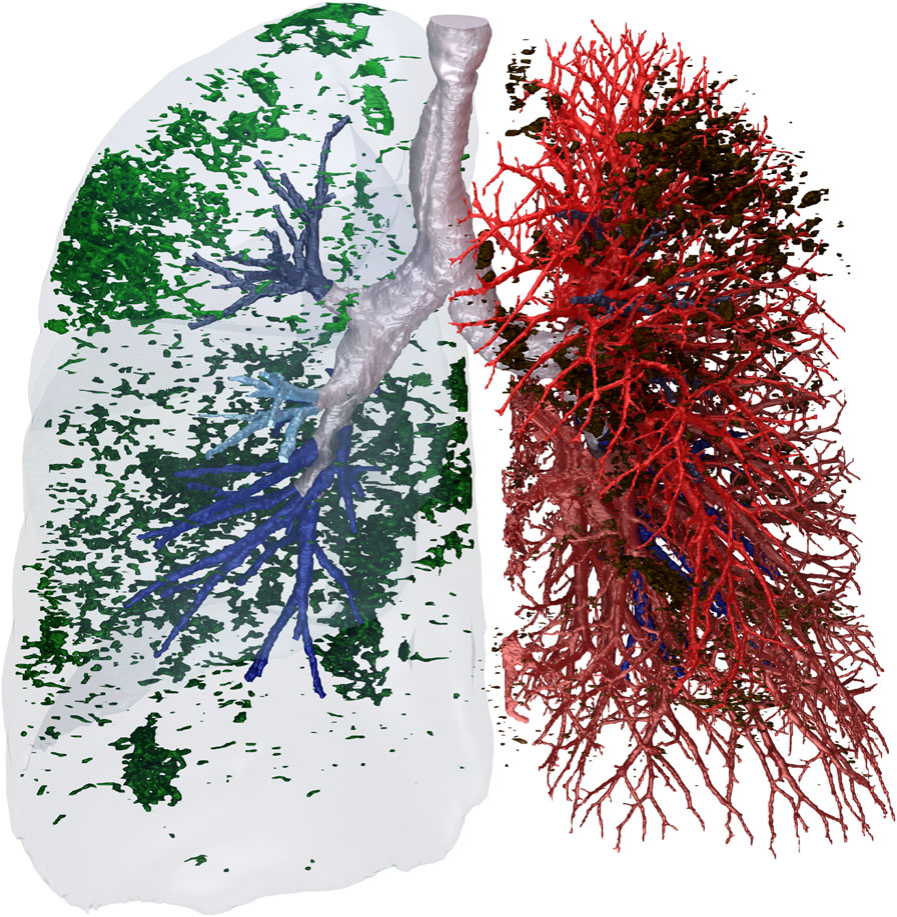
Functional Respiratory Imaging provides visualisation and quantification of airway volumes (depicted in blue), lobe volumes, fibrosis (depicted in green), emphysema (depicted in black) and blood vessel volumes (depicted in red)

Functional respiratory imaging (FRI) is a post-processing technology that utilizes multi-slice HRCT scans and computational fluid dynamics (CFD) to assess the overall lung health and function in a regional manner by quantifying endpoints as airway volume and resistance [22, 23]. FRI is considered a more sensitive method for observing changes in airway volume and resistance than classical lung function tests (e.g. forced expiratory volume in 1 s) [23–25]. This image-based method can also be used to provide a comprehensive assessment of airway tree changes [24, 26] (Fig. 1). Therefore FRI has the potential to better characterize disease, provide more accurate information in treatment follow-up of a patient in clinical practice and to predict and evaluate therapeutic interventions in many respiratory conditions. Comparable qCT methods to FRI have been used, in IPF and other fibrotic lung disease, as (treatment) endpoint [27–29].

The use of FRI and other qCT measurements may thus allow better monitoring of disease progression and response to treatment, improving our understanding of this disease.

## Methods

### Study design

The aim of this study was to identify FRI parameters, which predict FVC decline in patients with IPF. We retrospectively studied data from a Phase 2 open-label, dose-escalation study to evaluate the safety and efficacy of an anti-CTGF monoclonal antibody, pamrevlumab (FG-3019), for treatment of IPF. In the Phase 2 study, conducted by FibroGen, Inc., diagnosis of IPF was based on a usual interstitial pneumonia (UIP)-pattern on HRCT or a possible UIP-pattern with a UIP-pattern on surgical lung biopsy, as per applicable diagnostic guidelines at the time (2011) [2]. HRCT scans, with a breath hold at inspiration and used as outcome measure in the original study protocol, were taken at baseline, 24 weeks and 48 weeks after treatment commenced (*n* = 89 enrolled; 67 completed treatment; 66 full data set). The trial was composed of two dose cohorts. In the first, patients had been diagnosed with IPF within 5 years of trial inclusion with FVC ≥ 45% predicted and DL_CO_ ≥ 30% predicted and participants had to show disease progression in the last year (FVC decline ≥10%, HRCT worsening, and/or other objective changes). In the second cohort, the minimum FVC % predicted was raised to 55%. For all subjects, baseline HRCT had to indicate 10–50% reticular fibrosis and no more than 25% honeycombing. This study was performed in accordance with the Declaration of Helsinki. (www.ClinicalTrials.gov number NCT01262001) (Raghu Eur Respir J 2016; 47: 1481–1491) [29]. The investigators initiated this study in consultation with FibroGen, Inc. for use of the original HRCT data.

### Methods and analysis

Post-hoc analysis of this patient cohort was performed using FRI and mixed effects (regression) models on all available data in the data set, to understand change from baseline at week 24 and at week 48 for all patients in FRI parameters relative to FVC in terms of disease progression. Detailed explanation of this technique can be found in Additional file 1 and the FRI manual [30]. Subjects in this study had complete CT scans at baseline, week 24 weeks and week 48 weeks. FRI parameters were determined for the whole lung (all lobes), for the lower lung zones (right and left lower lobes) and for the upper lung zones (right upper and middle lobe; left upper lobe). Typical FRI parameters that were included were: specific image-based airway radius (siRADaw), percentage of fibrotic tissue at total lung capacity (TLC) and predicted lobe volume at TLC. In addition, sample size calculations were conducted to demonstrate sensitivity to change for each measurement (PFT or FRI parameter), from baseline to week 48. Sample sizes were obtained for a power goal of 80%, a significance level of 0.05 and two-tailed. These were based on the effect sizes calculated on the mean and standard deviation of the within subject differences between week 48 and baseline.

## Results

Patient characteristics are shown in Table 1. Subjects were predominately male with a mean FVC < 80% predicted.

**Table 1:** Patient characteristics

Number of subjects		89
Age [y] (range)		68 (47–82)
Male, n (%)		71 (79.8)
Time from IPF diagnosis, n (%)	< 1 year	34 (38.2)
	1–3 years	33 (37.1)
	> 3 years	22 (24.7)
FVC [L] (range)		2.52 (1.32–5.51)
FVC [% predicted] (range)		65.9 (42.6–111.7)

**Fig. 2:**
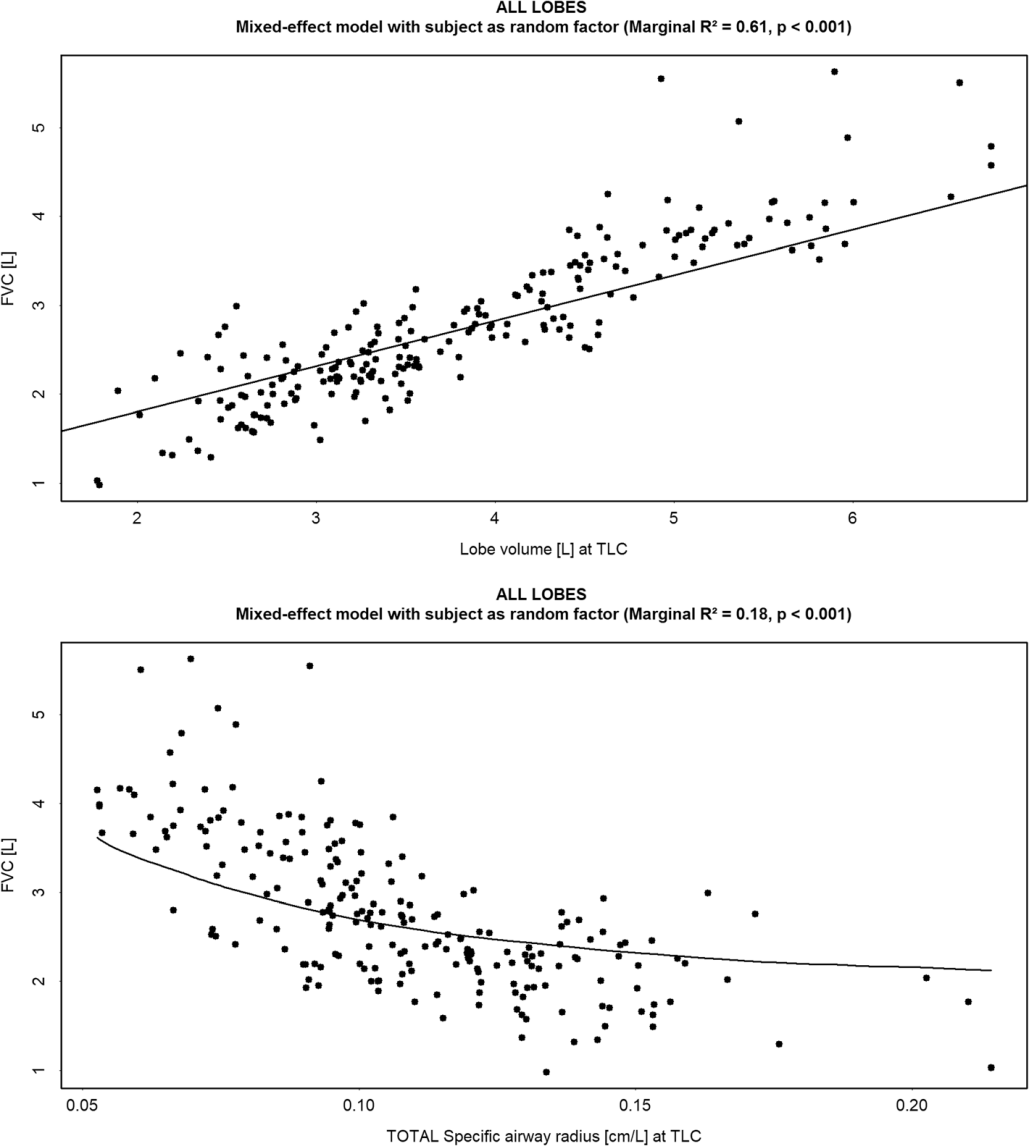
(*Upper panel*) Correlation between the FRI-based lung volume measured at Total Lung Capacity (TLC) [L] and the Forced Vital Capacity (FVC) [L]; (*Lower panel*) Correlation between the specific image based airway radius (siRADaw) measured at Total Lung Capacity (TLC) [cm/L] and the Forced Vital Capacity (FVC) [L]

**Fig. 3:**
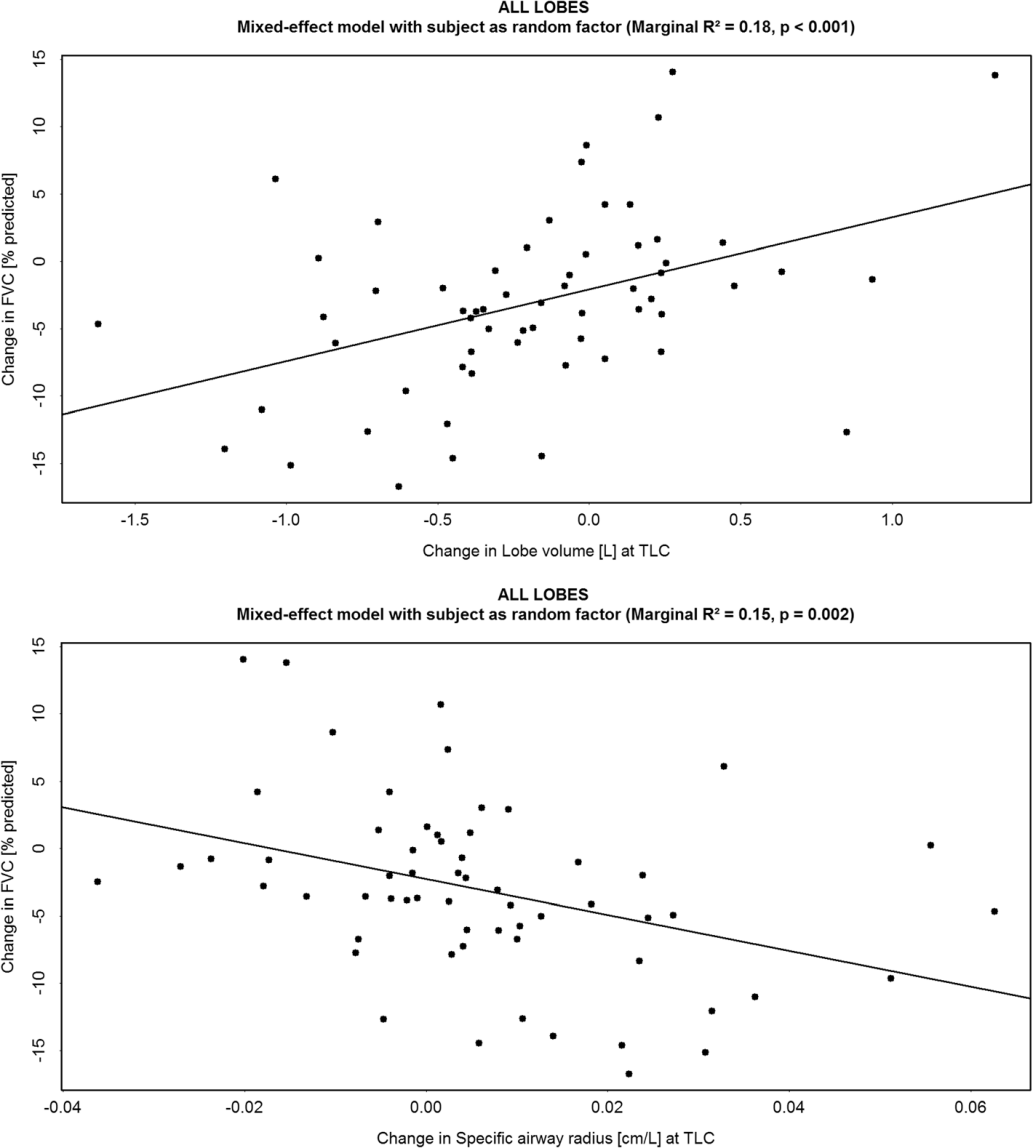
(*Upper panel*) Correlation between the change in lung volume measured at Total Lung Capacity (TLC) [L] and the change in Forced Vital Capacity (FVC) [% predicted]; (*Lower panel*) Correlation between the change in specific image based airway radius (siRADaw) measured at Total Lung Capacity (TLC) [cm/L] and change in FVC (% predicted)

**Fig. 4:**
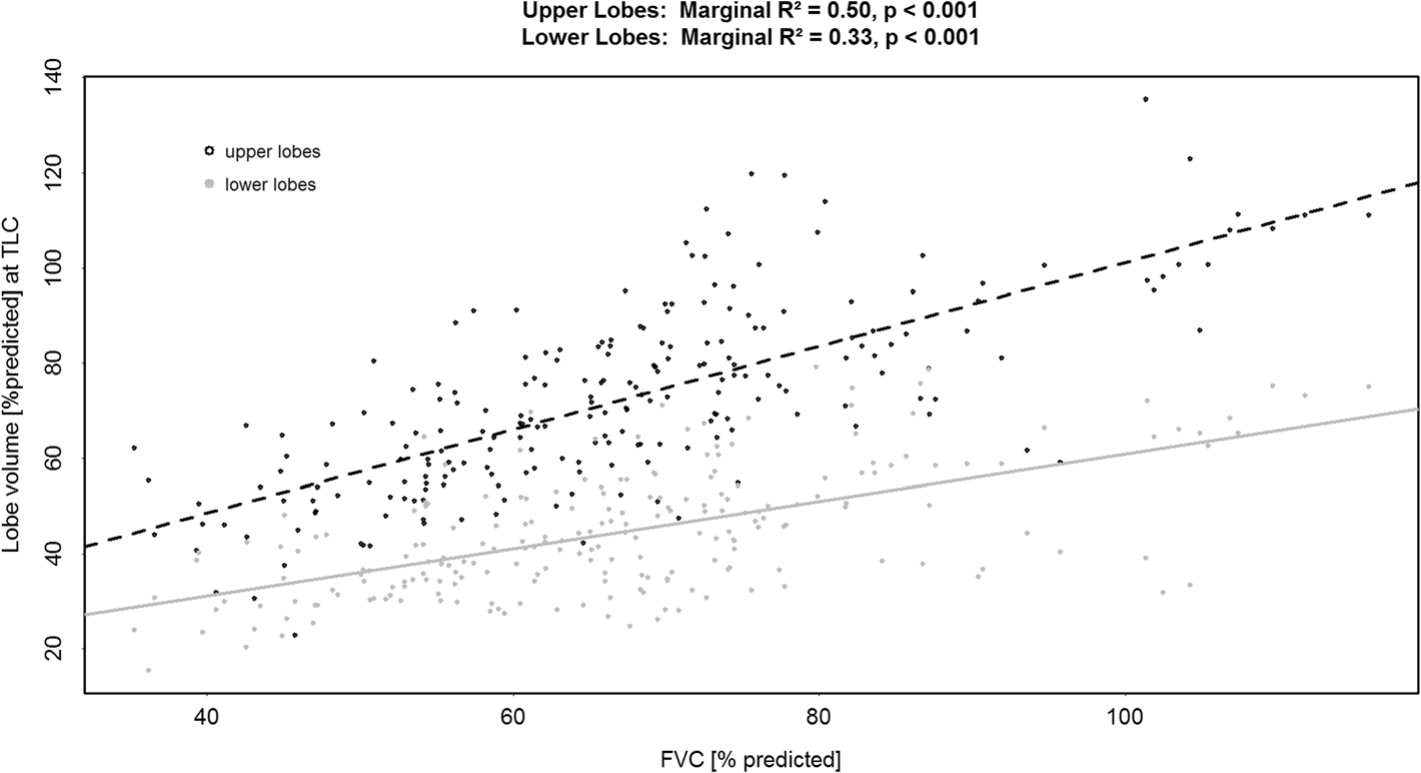
Correlation between Forced Vital Capacity (% predicted) and FRI-based lobe volume (% predicted) measured at Total Lung Capacity (TLC) for upper and lower lung zones

On univariate analysis, good correlation between lung volume based on FRI assessment and FVC was demonstrated (*R*^*2*^ = 0.61, *p* < 0.001) (Fig. 2). A negative correlation between specific airway radius and FVC was demonstrated at TLC (*R*^*2*^ = 0.18, *p* < 0.001) (Fig. 2). At TLC, FVC decline correlated significantly with lung volume decline (*R*^*2*^ = 0.18, *p* < 0.001) and increase in specific airway radius (*R*^*2*^ = 0.15, *p* = 0.002) (Fig. 3) both at TLC. The lower lobes were most affected (Fig. 4). Importantly, a decline in FVC was not observed until a 40–50% loss of lower lobe volume was measured.

**Fig. 5:**
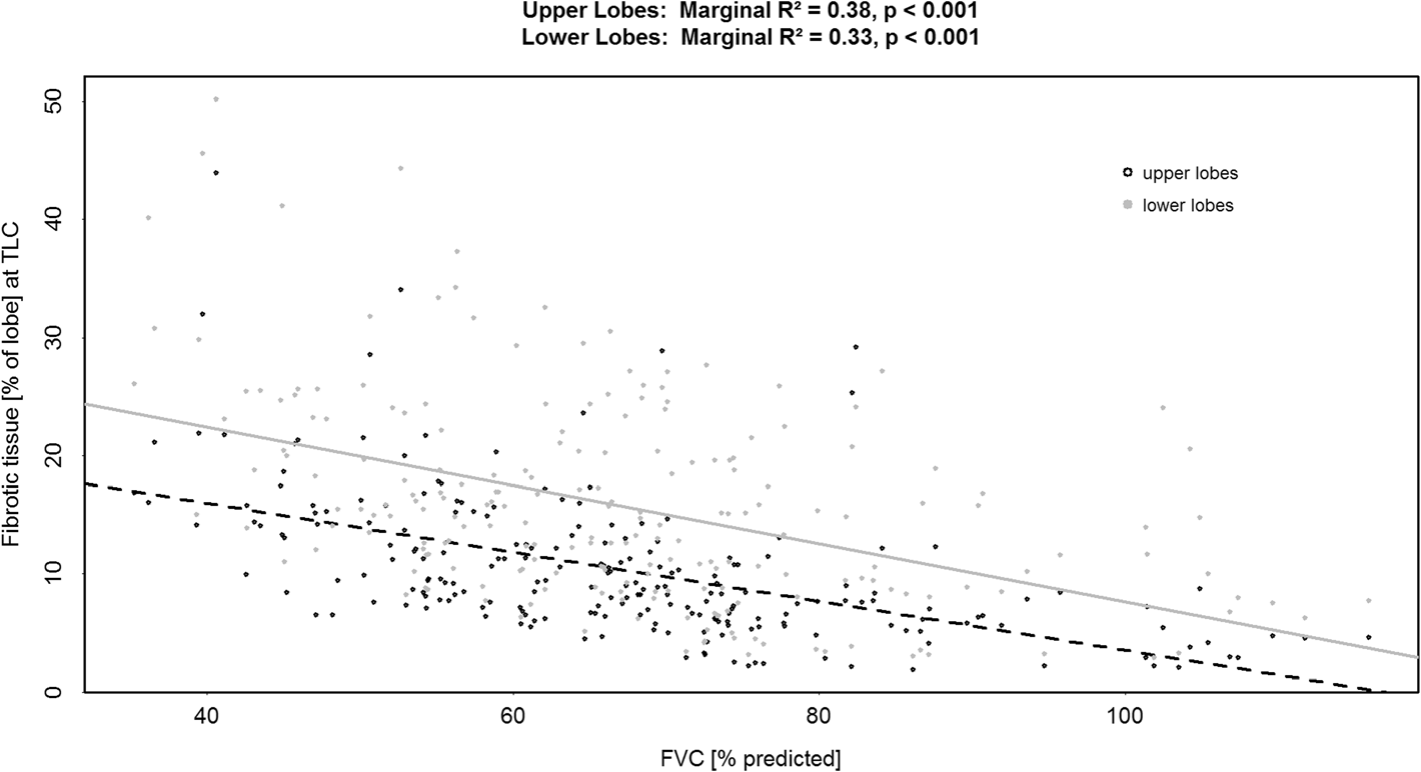
Correlation between Forced Vital Capacity (% predicted) and FRI-determined fibrotic tissue (% predicted) measured at Total Lung Capacity (TLC) for upper and lower lung zones. Fibrotic tissue is determined from the scans based on segmentation of areas with Hounsfield Units between − 600 and 600

In more advanced disease (i.e. lower FVC) there was a negative correlation with fibrotic tissue (Fig. 5). Again, the same observation can be made that the lower lobes are more diseased.

**Fig. 6:**
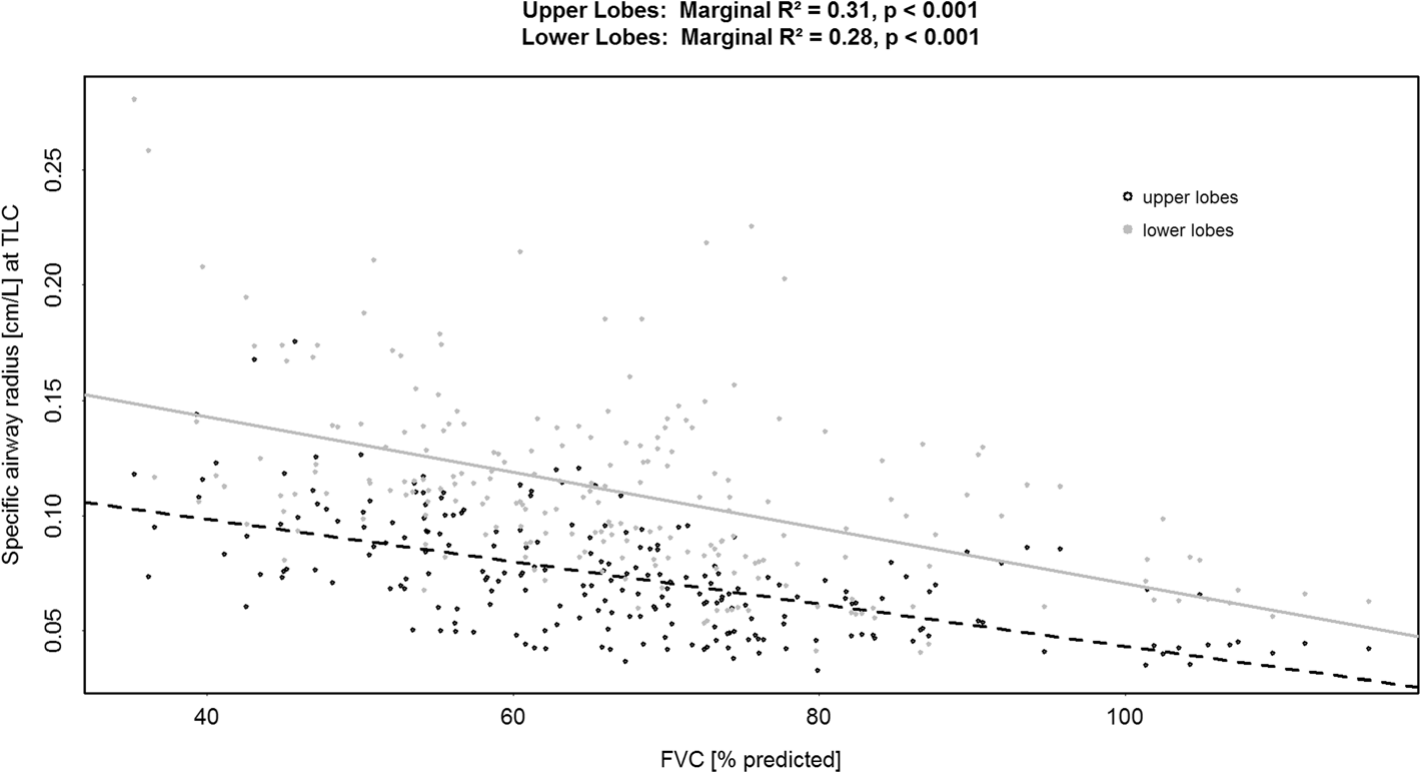
Correlation between Forced Vital Capacity (% predicted) and FRI-determined specific image based airway radius measured (siRADaw) at Total Lung Capacity (TLC) [cm/L] for upper and lower lung zones

Airway radius (expressed as siRADaw) increased with FVC decline (Fig. 6). This effect was more pronounced in the lower lobes. In contrast to observations made in the lobe volumes, there was a trend of divergence (regression lines) for a lower FVC, indicating that more pronounced disease correlated with larger airways relative to the total lung volume.

**Fig. 7:**
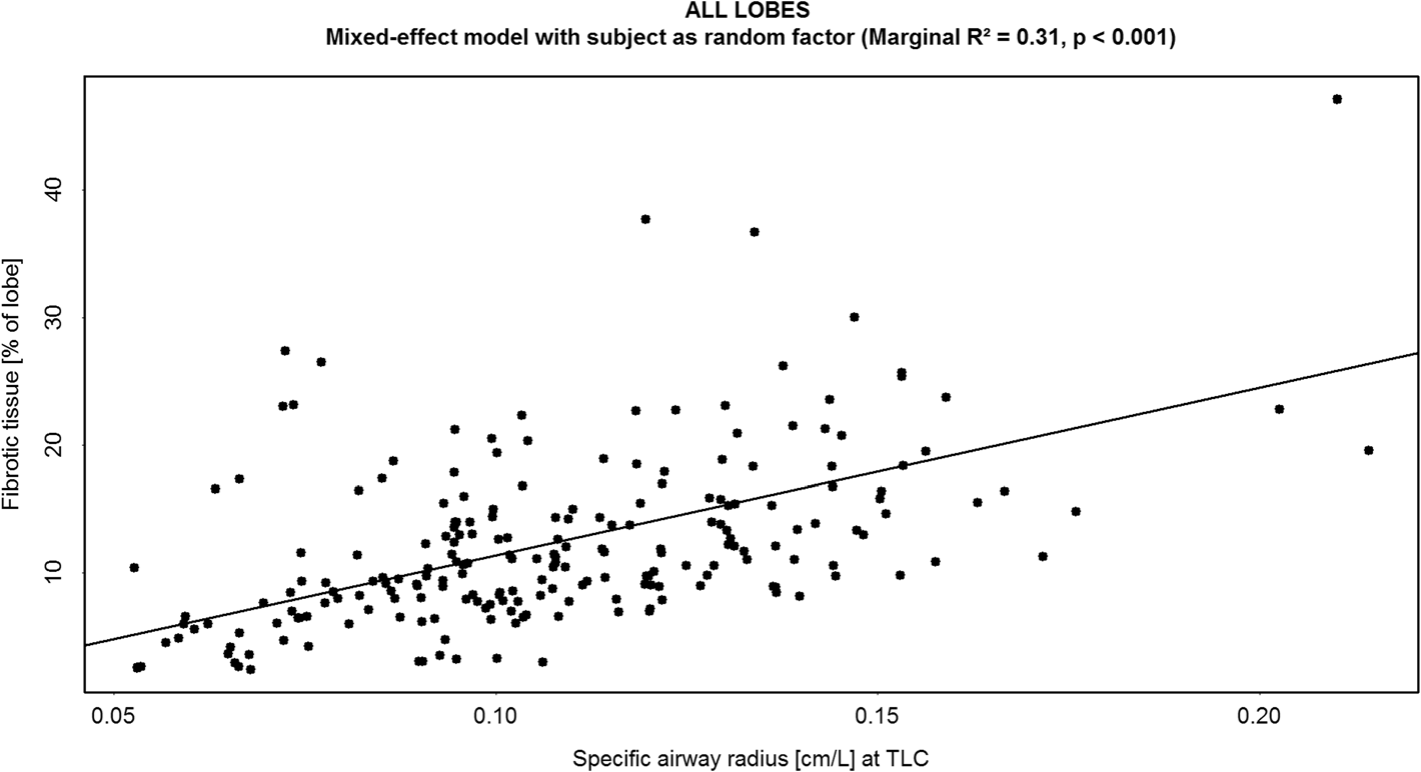
Correlation between the specific image based airway radius (siRADaw) measured at Total Lung Capacity (TLC) [cm/L] and FRI-determined fibrotic tissue (% predicted) measured at Total Lung Capacity (TLC)

Airway radius (siRADaw) also correlates positively with fibrotic tissue (*R*^*2*^ = 0.31, *p* < 0.001) (Fig. 7), i.e. airways enlarge, calculated in respect to the total lung volumes, with progressive disease.

Sample size calculations based on FRI parameters measured for the lower lobes were more sensitive in detecting change after 48 weeks (FVC: *N* = 43, effect size dz. = 0.437; lower lobe volumes: *N* = 42, effect size dz. = 0.443; lower lobe fibrosis: *N* = 28, effect size dz. = 0.549; lower lobe specific airway radius: *N* = 38, effect size dz. = 0.467). Moreover, when only considering patients with an FVC > 75% predicted at baseline, siRADaw lowered the required patient number from 101 to 13 patients (effect size dz. = 0.281 to 0.847) in demonstrating a significant change after 48 weeks. When assessing the changes after 24 weeks in this subgroup a sample size of 428 (effect size dz. = 0.136) was required when considering FVC as endpoint and 26 patients (effect size dz. = 0.572) for siRADaw endpoint. Dz stands for standardised difference scores, a technique typically used in within subject designs.

## Discussion

Our data shows a correlation between declining FVC with the FRI determined parameters: declining lung volumes, increasing fibrotic tissue and increase in the specific airway radius. For these three FRI parameters, the lower lobes are more affected even at the mild stage of the disease, keeping with early reports on IPF [31, 32].

IPF is a heterogeneous and unpredictable disease. The use of FVC is widespread in clinical research and practice, although the drawbacks and shortcomings of this test are well known [9, 10, 33, 34]. The use of qCT, as a new biomarker of disease characterisation, shows great potential in resolving the issue with FVC. Current qCT methods have focused on the lung parenchyma and pulmonary vessels. Many of these methods have great difficulty separating honeycombing from traction bronchiectasis and emphysema. Consequently, objective quantification of traction bronchiectasis severity, which has been reported as an important predictor of mortality in IPF, has been challenging [19–21, 35, 36].

FRI also captures lower lobe disease (50 to 60% predicted lobe volume) before any decline can be seen in FVC (100% predicted) [1, 2]. An explanation for this finding could be in the fact that FVC is patient effort dependent and is the sum total of everything that happens in the both lungs. The upper lobes likely compensate for the volume loss of the lower lobes. FVC remains fairly stable – or at least progresses slowly – until an FVC ± 75% predicted (Fig. 4) at which point the upper lobes also show progressive loss in volume and a more pronounced decline in FVC (convergence of the regression lines for the upper and lower lobes). This places the significant but weak correlations between FRI and FVC in perspective; that is, FRI already reveals disease related information not (yet) captured by the conventional lung function test. Similar correlations on (semi)-qCT for simultaneous changes in fibrosis and FVC have been reported [35, 37] with this exception that our data differentiates between upper and lower lobes and quantifies the loss of lobe volumes.

To the best of our knowledge we report, for the first time, the use of an automated method for quantification of traction bronchiectasis, overcoming the problems with semi-quantitative methods, which are inherently subjective and liable to significant interobserver variability. The airways are capturing disease signal that cannot solely be explained by the extent of disease itself. This is in keeping with a previous visual score study [38].

The observation that, progressive disease correlated with an increase in siRADaw (i.e. airway volumes), could likely be explained as a combination of two processes: traction bronchiectasis and the intra-pulmonary pressure re-distribution due to increased stiffness (resistance) of alveolar region.

**Fig. 8:**
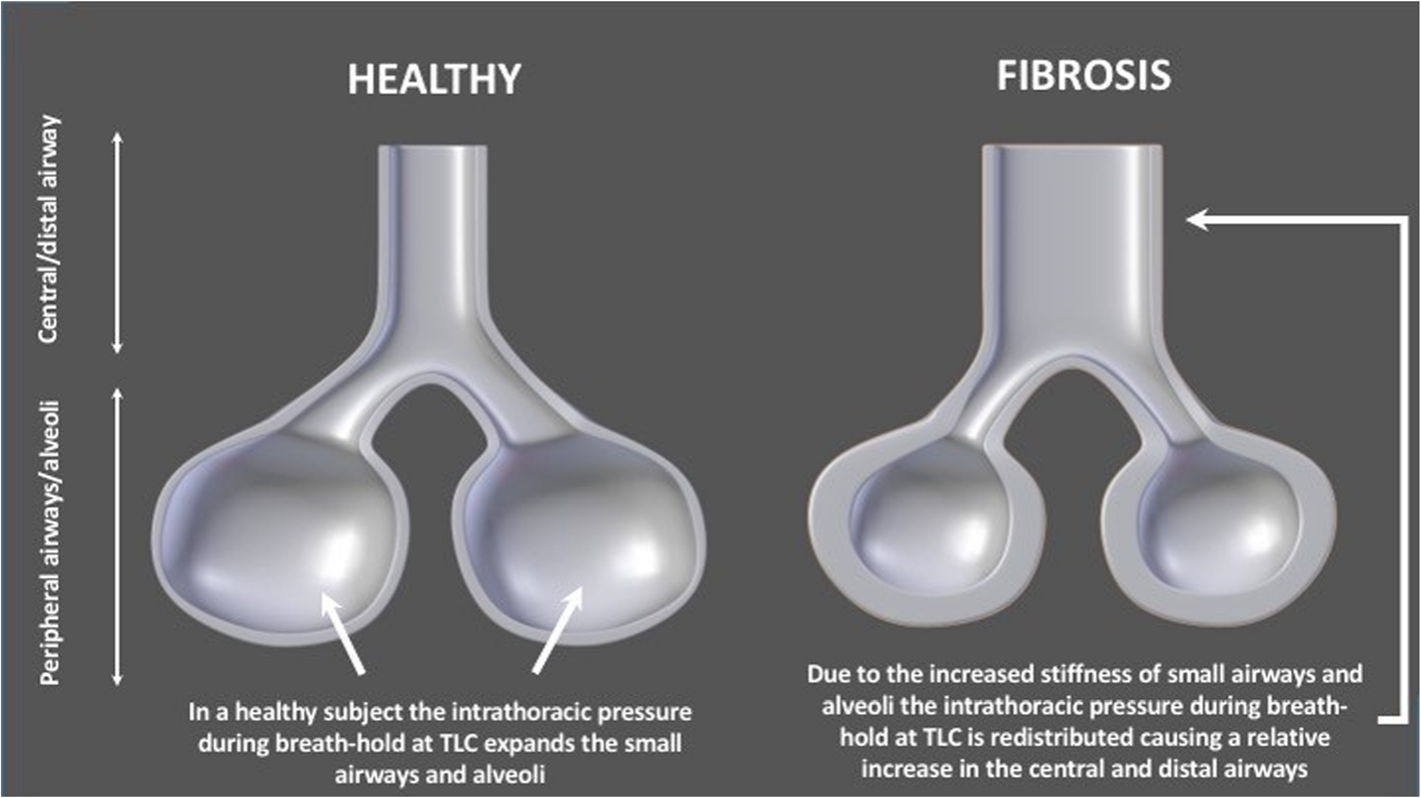
Hypothesis of IPF disease manifestation and progression in terms of FRI parameters. More severe IPF appears to be associated with relatively larger airways. This supports the rationale that the intra-thoracic pressure tends to redistribute due to the stiffness of the alveolar region and subsequently dilate the central and distal airways

In a CT scan taken at TLC during a breath hold, the intra-thoracic pressure tends to redistribute due to the stiffness of the alveolar region and subsequently dilates the central and distal airways as illustrated in Fig. 8. The relative enlargement of the airways is maintained and possibly exacerbated by traction bronchiectasis. The latter entails an increase in airway luminal dimensions due to the traction exerted by the fibrosis on the airway wall, as well as bronchiolar proliferation, both resembling disease progression in IPF [39].

HRCT findings of traction bronchiectasis correlate well with histopathology of fibroblast foci: profusion of fibroblastic foci is strikingly related to the severity of traction bronchiectasis [40, 41]. Traction bronchiectasis shows to be a clear indicator of mortality and remains a significant predictor of a poor outcome, independent of other associated parenchymal interstitial lung disease patterns [38, 42, 43]. Patients, recruited from the INPULSIS trials with possible UIP pattern on HRCT (i.e. traction bronchiectasis and no honeycombing), show to have the same grade of disease progression as a response to treatment with nintedanib in comparison to patients with definite UIP pattern (i.e. honeycombing) [44].

The specific airway radius may have a greater potential in predicting disease severity and progression than FRI-based lobe volumes alone, because of the increasing difference between the upper and lower lung zones in more advanced disease. Furthermore, siRADaw shows greater sensitivity for detecting change, especially in what are considered mildly diseased patients with an FVC > 75% predicted. Other IPF patient cohorts with mild disease eventually show progression [45, 46], thus regional information in IPF is clinically relevant and can be accurately captured using FRI.

Many patients with a FVC > 75% predicted demonstrated FRI characteristics associated with progressively declining FVC. This is an argument for the fact that early or asymptomatic disease is not detected by classical PFT measurements (i.e. FVC). Consequently, quantifying regional information about lung structures may reduce sample sizes needed to detect decline and treatment effect in IPF studies with new therapeutic options. Prospective validation of FRI use in IPF is warranted to, reliably, stratify patients in clinical trials as well as predicting outcome in individuals.

We acknowledge the fact that we didn’t compare our FRI measurements to a gold standard, although IPF clinical trial design has struggled the last decades to find a primary clinical endpoint (FVC or a qCT measurement) that can be routinely used with adequate precision [47, 48].

The correlation between decline in FVC and progressive reticular fibrosis (after 48 weeks) was also established in the original study cohort by a qCT method [29], as by another recent study [49]. This does demonstrate the utility and relevance of our qCT method (FRI) and accompanying results.

All patients studied in this trial were treated with a novel investigational drug (pamrevlumab) and no placebo arm was included in this study. The mixed effects model used in the statistical analysis of the data, however ensured that disease progression could be captured by using all available measurement points while correcting for multiple measurements per patient, thereby mitigating the potentially confounding effect of the treatment. The progression itself could potentially be influenced by a treatment effect so this IPF cohort does not represent natural disease progression.

## Conclusion

In conclusion, in patients with IPF, FRI parameters (siRADaw, percentage of fibrotic tissue at TLC and predicted lobe volume at TLC) allow monitoring of regional changes in disease and may capture disease progression in patients with preserved FVC.

## Additional file

Additional file 1: Appendix - Functional respiratory imaging (FRI) methodology. Detailed description of the FRI methodology and FRI parameters used in the manuscript/study. (PDF 74 kb)

## Abbreviations

(HR)CT: (High resolution) computed tomography
CFD: Computational fluid dynamics
FRI: Functional respiratory imaging
FVC: Forced vital capacity
IPF: Idiopathic pulmonary fibrosis
iVlobe: Imaging lobar volume
PFT: Pulmonary function test
qCT: Quantitative computer derived computed tomography
siRADaw: Specific image based airway radius
TLC: Total lung capacity
UIP: Usual interstitial pneumonia
