# Supplementary material for: Quantitative CT analysis using functional imaging is superior in describing disease progression in idiopathic pulmonary fibrosis compared to forced vital capacity
Source: Respir Res. 2018 Nov 6;19:213. doi: 10.1186/s12931-018-0918-5 (PMC6218992; doi:10.1186/s12931-018-0918-5)
Supplement: Supplementary file 1 — Appendix - Functional respiratory imaging (FRI) methodology. Detailed description of the FRI methodology and FRI parameters used in the manuscript/study. (PDF 74 kb) [file 12931_2018_918_MOESM1_ESM.pdf]

Appendix

Functional respiratory imaging (FRI) methodology

Regarding quantitative CT Imaging Processing, CT scan data was converted into 3D models of airways and lung lobes using Mimics (Materialise, Leuven, Belgium) a commercially available validated software package (Food and Drug Administration, K073468; Conformité Européenne certificate, BE 05/1191.CE.01). Other software used included; TGrid 14.0 (Ansys Inc, Canonsburg, PA) for 3D meshing and Fluent 14.0 (Ansys Inc, Canonsburg, PA) for CFD simulations.

Segmentation of the tracheobronchial tree was done using directional thresholding with automated leakage detection. Automatic airway segmentation was performed up to the point where no distinction could be made between the intra-luminal and alveolar air. Following automated segmentation of the bronchial tree, the airways were manually checked. Missing branches were added to the bronchial tree and incorrect branches were deleted when necessary. The respiratory tract was reconstructed down to the level of airways with a diameter of 1–2 mm, beyond this point, the HRCT resolution is insufficient to distinguish alveolar from intraluminal air. The segmented airway tree was converted into a 3D model that was smoothed using a volume compensation algorithm. The smoothed model was trimmed perpendicular to the airway centreline at the trachea (using the middle point of the superior side of the sternum as a landmark) and at each terminal bronchus. Remaining artefacts due to noise in the CTs were then manually removed from the model. Finally a series of manual quality checks were performed. Total time for the automated steps and manual quality checks varied from 2 to 6 hours per scan.

FRI is comprised of a combination of airway segmentation, lung volume segmentation by lobe, and airway resistance calculations based on computational flow simulation using boundary conditions provided by the lobe expansions from functional respiratory capacity (FRC) to total lung capacity (TLC), allowing calculation of imaging lobar volume (iVlobe), imaging airway volume (iVaw) and imaging airway resistance (iRaw), as well as their specific values (corrected for lobe volume). Specific image based airway radius (siRADaw) is the radius

derived from the segmented airway volume (iVaw) normalized by the lung volume, hence made specific The airway radius itself (iRADaw) is calculated based on segmented airway volume and airway surface The airways were subsequently divided in central and distal regions. The central airway volume is defined as the region from the trachea to the segmental bronchi (the combination of the trachea, main bronchus left, main bronchus right, truncus intermedius, right upper lobe bronchus, right middle lobe bronchus, right lower lobe bronchus, left upper lobe bronchus, superior division bronchus, lingular bronchus, left lower lobe bronchus). The distal airway volume is defined as the segmented airway volume starting from the 3rd bifurcation (4th generation), which include segmental bronchii (B1R, B2R, etc.) and subsegmental bronchii that are discernible on the scan (bronchii with a diameter > 1-2 mm). iVlobe from the CT images at RV (e.g. expiratory CT) and TLC (e.g. inspiratory CT) were also extracted. By means of application of CFD on the segmented airway model, iRaw were calculated. Lungs were split into lobes by identification of the fissure lines from the CT scan. This allowed determination of total lung volume and of the volume of each lobe individually. Lobar % predicted values are derived from reference values for total lung capacity as a function of age, gender and height in combination with lobar volume distribution as derived from HRCT scans from healthy volunteers, based on Quanjer ERS '93 reference equations [1]. In CT scans without the use of a contrast agent, blood vessel volume (iVbv) is used for indicating fibrotic tissue progression. A fibrotic score was computed from the TLC scan based on a Hounsfield Unit range of [-600,600].

[1] Quanjer PH, Tammeling GJ, Cotes JE, Pedersen OF, Peslin R, Yernault JC. Lung volumes and forced ventilatory flows. Report Working Party Standardization of Lung Function Tests, European Community for Steel and Coal. Official Statement of the European Respiratory Society. Eur Respir J Suppl. 1993;6(suppl 16):5-40
